# Half a century of handedness research: Myths, truths; fictions, facts; backwards, but mostly forwards

**DOI:** 10.1177/2398212818820513

**Published:** 2019-05-06

**Authors:** Chris McManus

**Affiliations:** Research Department of Clinical, Educational and Health Psychology, University College London, London, UK

**Keywords:** Handedness, lateralisation, brain asymmetry, cerebral specialisation, genetics

## Abstract

Although most people are right-handed and have language in their left cerebral hemisphere, why that is so, and in particular why about ten per cent of people are left-handed, is far from clear. Multiple theories have been proposed, often with little in the way of empirical support, and sometimes indeed with strong evidence against them, and yet despite that have become modern urban myths, probably due to the symbolic power of right and left. One thinks in particular of ideas of being right-brained or left-brained, of suggestions that left-handedness is due to perinatal brain damage, of claims that left-handers die seven years earlier than right-handers, and of the unfalsifiable ramifications of the byzantine Geschwind-Behan-Galaburda theory. This article looks back over the past fifty years of research on brain asymmetries, exploring the different themes and approaches, sometimes in relation to the author’s own work. Taking all of the work together it is probable that cerebral asymmetries are under genetic control, probably with multiple genetic loci, only a few of which are now beginning to be found thanks to very large databases that are becoming available. Other progress is also seen in proper meta-analyses, the use of fMRI for studying multiple functional lateralisations in large number of individuals, fetal ultra-sound for assessing handedness before birth, and fascinating studies of lateralisation in an ever widening range of animal species. With luck the next fifty years will make more progress and show fewer false directions than had much of the work in the previous fifty years.

The fact that most people are right-handed is immediately obvious from everyday experience. Handedness has been recorded in written records since the time of antiquity (McManus, 2002), artistic portrayals show a predominance of right-handers for at least 5000 years (Coren and Porac, 1977), and it is probable that as a species most humans have been right-handed for several million years (McManus, 2009), although other animals show something much closer to a 50:50 right–left split. The history of left-handedness is less clear, but archaeological data on Neanderthals suggest left-handers have existed for half a million years or so (Frayer et al., 2012).

Handedness as a topic of scientific research has a rather shorter history, and to a large extent it fits both within the 50-year lifespan of the BNA, which mostly coincides with the 4.5 decades since I began exploring lateralisation (McManus and Humphrey, 1973). This is therefore an intellectual and a personal history. As an intellectual history, it cannot be comprehensive, and so the reader wanting a general overview is directed to Clare Porac’s (2016) *Laterality: Exploring the Enigma of Left-Handedness*, with its sub-title emphasising how much is still to be understood. Here, I mainly consider research originating in what historians might call the five ‘long decades’ (10 ± 5 years), research which illustrates the range of issues, some of the successes and some of the failures.

Handedness perhaps has had more than its fair share of false and misleading ideas, which come under the broad heading of ‘neuromythology’ (Tallis, 1991), for, as Mike Corballis (1980) said, laterality research can be ‘stalked by the demons of mythology’. The myths often originate in the symbolism which pervade asymmetry, with ‘right’ universally seen as ‘good’ and ‘left’ as ‘bad’. A broader understanding of the symbolic meanings of handedness, later called ‘dual symbolic classifications’ (Needham, 1973), was first properly explored in Robert Hertz (1909) anthropological study *La prééminence de la main droite: étude sur la polarité religieuse* (Hertz, 1909), translated as *Death and the Right Hand* by Rodney and Claudia Needham (Hertz, 1960), with the broader meanings of handedness well summarised in Wile’s (1934) *Handedness: Right and Left* (Wile, 1934).

Understanding handedness is, of course, closely related to understanding brain lateralisation, and the two inevitably overlap. Measuring brain lateralisation directly is still not easy, and handedness continues to be a cheap, easy and reliably measured surrogate for brain lateralisation. To understand handedness properly would probably also be to understand cerebral lateralisation properly, with all its implications for understanding human evolution and pathology.

## Before the 1970s

Handedness was studied surprisingly little in the 19th and early 20th centuries, although it was always lurking on the margins. The corollary of Dax and Broca’s realisation that most people had language in the left hemisphere was that some people had language in the right hemisphere (Harris, 1993a). The too easy assumption that left-handed aphasics would all have right-sided lesions took surprisingly long to refute (McManus, 1983). Still perhaps the most tantalising handedness statistic is that about 5%–6% of right-handers have right-hemisphere language along with only about 30% of left-handers, the numbers not being easy to explain.

The modern interest in handedness research in neuroscience probably originates in studies on speech and handedness by Oliver Zangwill (1955, 1960), followed by Hécaen and de Ajuriaguerra’s (1964) *Left-handedness: Manual Superiority and Cerebral Dominance*, with the dichotic listening studies of Doreen Kimura (1961) and Phil Bryden (1962) opening the path to studying the relation of handedness and cerebral lateralisation in normal populations. The effects of handedness on other behaviours were developed in Margaret Clark’s (1957) *Left-Handedness: Laterality Characteristics and Their Educational Implications.*

The early history of handedness was chequered and diffuse. A few themes were recurrent, particularly that handedness might have a genetic basis, with family studies by Ramaley (1913), Chamberlain (1928), Rife (1940), and Trankell (1955) suggesting handedness ran in families, but that was countered by studies of twins finding a disconcerting lack of handedness concordance in identical twins (Newman et al., 1937; Wilson and Jones, 1932; Zazzo, 1960). Throughout the years, there have therefore been recurrent suggestions that handedness was learned as a result of environmental factors, following Plato, who blamed right-handedness on the social pressure of ‘nurses and mothers’ (Laws, 794a). That social pressure does sometimes occur is undoubted, as shown in the attempts during the 19th century to force left-handed children to be right-handed (Ireland, 1880), with such efforts encouraged in part by the unsubstantiated claims of Cesare Lombroso (1903) of left-handedness being associated with criminality.

Asymmetries are found not only in the human brain but throughout the biological and physical world, with ‘handedness’ used as a rather loose descriptor. Many asymmetries were described in Ludwig’s (1932) encyclopaedic review *Das Rechts-Links-Problem im Tierreich und beim Menschen*. Asymmetries at the biochemical level had of course been first described by Louis Pasteur in 1848 and chirality rapidly became well understood in organic chemistry. Finally, asymmetry at the sub-atomic level erupted into in public consciousness in 1957 after the physicists Chen Ning Yang and Tsung-Dao Lee won the Nobel Prize for their astounding discovery of asymmetries of the weak force, a failure of parity conservation. The results were later popularised, along with accounts of many other asymmetries, in Martin Gardner’s (1990) *The Ambidextrous Universe*, first published in 1964.

## The long 1970s

Like all of the decades, the 1970s saw both progress and false leads. Empirically, as perhaps is ever the case, there is nothing so practical as a simple measuring instrument, and the Edinburgh Handedness Inventory, described in one of his last papers by Carolus Oldfield (1971), has swept the board. Although hardly the first or last such questionnaire – Oldfield describes four predecessors – it is by far the most widely used instrument, albeit its original eccentric response scheme is mostly replaced by a conventional five-point scale (Edlin et al., 2015).

Integrating both theory and experiment, Mike Corballis (2017), who continues to publish on the evolution of language, was involved in two major theoretical studies. *The Psychology of Left and Right* by Corballis and Beale (1976) was an experimental psychological approach to answering subtle questions about perceiving right and left, going back to the key insight of Ernst Mach (1914) that truly perceiving left and right requires an asymmetric brain.

Two years later, Corballis and Michael Morgan wrote two long, authoritative, wide-ranging papers considering the deep biological underpinnings of human and animal laterality, in a framework of universal left–right gradients in biology (Corballis and Morgan, 1978; Morgan and Corballis, 1978). Although they were correct in asserting that genes are agnosic (‘genes do not, perhaps cannot, encode the direction of a structural asymmetry’), they probably erred in concluding that ‘There is … little evidence that genetic variation plays any significant role in causing variations in human laterality’ (Morgan and Corballis, 1978: 276). Nevertheless, the papers kick-started biological research into handedness.

A further theoretical insight from the long 1970s was Paul Satz’s (1972) important concept of pathological left-handedness, which had a simple but powerful insight. If an asymmetric brain is damaged by random, and hence *symmetric*, processes, then the outcome will not be symmetric, more right-handers becoming left-handed than left-handers becoming right-handers, so that pathological handedness will be more frequent in left-handers than in right-handers. The model is a valid one but rapidly was extrapolated too far. A general belief at the time in the role of environmental factors over genetic factors (as also occurred in the contemporary ‘IQ wars’ (Kamin, 1974)) resulted in Bakan’s (1971, 1975) and Bakan et al.’s (1973) influential claims that left-handedness resulted primarily from pathological factors, with obstetric trauma in particular causing minimal brain damage. The data were almost entirely indirect (birth order effects, self-recall of maternal birth history, etc.), but the idea was influential enough for my own PhD thesis to start by analysing it. After using similar questionnaire-based methods to Bakan, my personal breakthrough came from discovering the very large dataset of the 1958 National Child Development Study (NCDS), the second of the very large cohort studies which particularly characterise British medical and social research (Pearson, 2016). Not only were midwife-recorded birth details recorded for 11,000 births, but handedness was recorded by professionals at age 7 and 11. More remarkably, the data were freely available and arrived in my pigeon-hole as two large magnetic tapes. The data were clear – left-handedess showed minimal or probably no relationship to birth trauma (McManus, 1979, 1981). Perhaps one of the first handedness studies to use a major cohort study, handedness is now available in a range of large epidemiological and social studies and such studies have clarified many aspects of handedness.

The idea that the majority of left-handers were left-handed due to pathological factors was revoked by Dorothy Bishop’s (1990) studies of right- and left-hand motor control which suggested that at most about one in 20 left-handers may have a pathological aetiology.

For serious false leads in lateralisation, one has to look no further than the idea of ‘brainedness’, that most people use only one hemisphere for thinking, the left if they are scientific and the right if they are artistic, with left-handers inevitably being more right-brained or even using both hemispheres. Supported by studies using measures of creativity, the entire research enterprise now looks embarrassingly like a hangover of flower-power and the 1960s. Certainly when we reviewed it, we could find little support (Beaumont et al., 1984) although the idea continues to rumble on in popular literature, finding grand, if sometimes grandiose, expression in *The Origin of Consciousness in the Breakdown of the Bicameral Mind* of Julian Jaynes (1976), and much later perhaps in *The Master and His Emissary: The Divided Brain and the Making of the Western World* of McGilchrist (2009). The idea of brainedness, as with much neuromythology, has failed to die, with 64% of the general public still agreeing with the statement that ‘Some of us are “left-brained” and some are “right-brained” and this helps explains differences in how we learn’ (Macdonald et al., 2017).

A second false lead was probably that of Geschwind and Levitsky (1968), who showed in post mortem studies that the planum temporale was on average larger in the left hemisphere, and since language is mostly in the left hemisphere, they concluded that the planum temporale was responsible for language dominance. Although much work has been carried out, the picture is still, at best, far from clear (Shapleske et al., 1999), despite large numbers of careful measurements.

The long decade also saw Michael Barsley’s (1966, 1970) popular books on left-handedness, with their myriad successors in books, newspapers and on the Internet, which also promulgated a series of endlessly repeated half-truths and myths (Elias, 1998).

## The long 1980s

Measurement continued as an important theme in the long 1980s. The Annett pegboard, first used in the 1970s and the basis of an important series of papers over the next two decades, provided the first standardised task of lateralised motor performance (Annett, 1970). The pegboard study carried out jointly by John and Marian Annett (Annett et al, 1979) also provided a rare analysis of the kinematics of right and left hands. The Annett pegboard is striking for its unimodal, near normal, distribution, which later became a key feature of Annett’s genetic model. In the 1980s, other researchers followed Annett’s pioneering work measuring motor performance directly, but the important feature of the rapid tapping task of Peters (1980) and Peters and Durding (1978) and the circle-marking task of Tapley and Bryden (1985), the latter allowing group administration, was the overall bimodal distribution with little overlap of right and left-handers. The difference between the unimodal and bimodal distributions is probably explained by the greater measurement error of the pegboard, the minor distribution sliding under the major, as was suggested at the time (Annett, 1985b; McManus, 1985b, 1985c), and continues to be studied (Bryden and Roy, 1999; McManus et al., 2016).

The end of the decade saw another important finding on motor skill by Peters (1990) that writing hand and throwing hand could be discordant in left-handers, a third of left-handed writers throwing more accurately with the right hand; subsequently the discordance was also found in right-handers, although only 3%–5% of right-handed writers throw better with the left hand (McManus et al., 1999).

The 1980s saw a resurgence of interest in the genetics of handedness. Earlier genetic models of handedness had typically involved one or two genes (Annett, 1964; Chamberlain, 1928; Jordan, 1911; Levy and Nagylaki, 1972; Ramaley, 1913; Rife, 1950; Trankell, 1955), usually with one gene labelled ‘Right’ and the other ‘Left’, which ran counter to Morgan and Corballis’ important idea of genes being left–right agnosic. Most genetic models had problems with the low rate of left-handedness in the children of two left-handed parents and foundered almost entirely on the low concordance of identical twins. Genetics does not however say that identical twins should be identical but rather that identical twins should be *more similar* than non-identical twins. A review of twin studies showed that to be the case (McManus, 1980), and it was reconfirmed by two later meta-analyses (Sicotte et al., 1999; Medland et al., 2006). The two competing models of the long 1980s, the ‘right-shift model’ of Annett (1978, 1979, 1985a) and my own DC model (McManus, 1979, 1984, 1985a), both accounted fairly well for twin data, family data and the relationship of language dominance to handedness. Both models succeeded as a result of having a large random component, ‘fluctuating asymmetry’ as biologists called it (Palmer and Strobeck, 1986), as if a coin were being tossed during development in some cases, the randomness explaining monozygotic twin discordance. The random component was compatible with data studying the side of the heart in mouse breeding experiments, where the ‘*iv*’ (inversus) gene also showed a strong random component (Hummel and Chapman, 1959; Layton, 1976). The two similar but different genetic models of handedness, Annett and McManus, produced an inevitable debate between the two proponents, both in the genetics and the phenotypics, which may have confused those outside the dispute. While there may have been heat as well as light, like all scientific arguments, the advantage was beneficial in forcing clearer theoretical analyses and statements of the models. These and other models were compared and contrasted at the end of the long 1980s (McManus and Bryden, 1992).

A false lead in the 1980s was the massive and influential work of Geschwind, Behan and Galaburda. The original paper of Geschwind and Behan (1982) was short and incisive, with the remarkable claim that left-handedness was associated with a range of diseases, particularly auto-immune conditions. Two years later, and a few months after Geschwind’s death, followed three massive papers by Geschwind and Galaburda (1985a, 1985b, 1985c), which later were reprinted as a book, *Cerebral Lateralization: Biological Mechanisms, Associations, and Pathology* (Geschwind and Galaburda, 1987). Almost overwhelming in its massing of evidence, the variety of theoretical mechanisms and the ability apparently to explain a vast, disparate array of phenomena, the central claim was that variation in foetal levels of testosterone explained many phenomena to do with brain lateralisation, which can now be seen as part of a more general over-emphasis on the size of sex differences and the role of testosterone (Fine, 2017). The paper was immediately controversial; Robert Joynt (1985) in an editorial in the journal where it was published described it as ‘speculative … bold … provocative’, with the editorial board ‘not in total agreement’. Making sense of the theory was far from straightforward, and so Phil Bryden and I first published a clear description of what the model seemingly said (McManus and Bryden, 1991). A year or two later, in conjunction with Barbara Bulman-Fleming, followed an empirical review of the support for the model, particularly concentrating on the central, and a *priori* most unlikely, claim that left-handedness was associated with auto-immune disorders. A meta-analysis found the evidence to be extremely weak (Bryden et al., 1994a, 1994b). Despite various criticisms, inevitably the theory has rumbled on until the present day, with claims still being made for it. The theory had the beneficial effect of forcing attention on handedness, but the strange, sometimes wild, claims resulting from research were probably in most cases the results of publication bias. It was a decade or two before foetal testosterone could be measured directly, with final nails in the coffin coming from findings of no relation between foetal testosterone levels and handedness or brain lateralisation (Grimshaw et al., 1995; Pfannkuche et al., 2009).

## The long 1990s

If theorising dominated handedness research in the 1980s, the long 1990s was a period of consolidation, with empirical data in much greater amounts in more solid form. Publication of research was probably helped by the 1996 launch of the journal *Laterality*, edited by Bryden, Corballis and McManus, with Michael Peters coming in after Bryden’s sudden death in 1996. The journal specialised in the study of lateralisation, providing a home for what previously had been a diffuse, scattered literature which lacked a focus. A special triple issue of *Laterality* in 2016 was devoted, two decades after his death, to the enduring legacy of Phil Bryden, who influenced so much research from the 1960s to the 1990s, including reminiscences by the present author (Corballis et al., 2016). The special issue was edited by Daniel Voyer and Gina Grimshaw (2016), two of the many talented research students whom Phil supervised.

Technological advances benefitted laterality research, as with all areas of psychology and neuroscience, with one unexpected source being routine, real-time ultrasound scanning of foetuses during pregnancy. An important series of papers by Hepper et al. (1990, 1998, 2005) and Hepper (2013) showed that most but not all second and third trimester foetuses preferentially sucked the right thumb, those sucking the right (left) thumb subsequently becoming right (left) handed. Asymmetries of limb movements were also found in first trimester foetuses, long before cortical connections to the limbs have developed. These remarkable findings not only emphasise the prenatal origins of handedness (and definitively reject Plato’s emphasis upon the role of mothers and nurse maids) but also exclude a majority of hypothesised, postnatal, environmental processes that have been speculated on as causes of handedness. That pushed the emphasis once more upon genetic factors, although little of major substance happened again until the 2000s.

In many ways, the most important advance of the 1990s was integrative, particularly with a meeting organised by the Ciba Foundation in London in February 1991, entitled *Biological Asymmetry and Handedness* (Bock and Marsh, 1991). The key instigators were Lewis Wolpert and Nigel Brown, developmental biologists who had developed the influential ‘F model’ (Brown and Wolpert, 1990) of how anatomical asymmetries might develop during embryogenesis. The result was an intense, discussion-full, interdisciplinary, 3-day meeting with physicists, biochemists, microbiologists, zoologists, palaeontologists and neuroscientists, including ‘the three Michaels’ (Corballis, Morgan and Peters), Marian Annett, Tim Crow, Albert Galaburda and myself. Throughout, there was a growing awareness not only of potential overlaps between the fields but the likelihood of common processes beneath the disparate biological and neuroscientific phenomena. Much was hope and dreams, but biological reality appeared just 4 years later with a crucial paper published by Mike Levin and Cliff Tabin (Levin et al., 1995), which showed that a key biological lateralisation, the side of the vertebrate heart, usually on the left (*situs solitus*), was determined in the chick by the early asymmetric expression of three genes, *activin, nodal* and *sonic hedgehog*. Most dramatically, a bead coated in *activin* or *sonic hedgehog* protein placed on the right-hand side of a very early, symmetric, chick embryo induced the heart to be on the right-hand rather than the left-hand side (*situs inversus*).

Elegant embryology subsequently worked out the underlying cascade of processes which ultimately ended up as visceral asymmetries. However, the symmetry-breaking event itself was still not yet clear, although for a number of years it had been known that humans with PCD (primary ciliary dyskinesia) had defects of ciliary movement in lungs and sinuses, which resulted in the strange triad of chronic sinusitis, bronchiectasis and *situs inversus*, with the latter present only in half of the cases (Afzelius, 1979; Kartagener, 1935; Siewert, 1904). The *iv* mutation in mice had also been known for 50 years to show similar properties, 50% of homozygotes having *situs inversus* (Hummel and Chapman, 1959). The hunt had therefore long been on for the mechanism underpinning *situs inversus* in the mouse and the remarkable breakthrough came in a series of papers from the laboratory of Nobutaka Hirokawa, in Tokyo. The early mouse embryo, at the end of the gastrula stage, consists mostly of a ball of visually undifferentiated cells with only the primitive streak visible. The nodal region, a rhomboidal area, appears transiently at its anterior end, and in its base are cilia, the function of which had not been known. The key observation was that these cilia all rotate in the same direction, forcing extracellular fluid from right to left, that in turn caused the development of typical *situs solitus* (Nonaka et al., 1998). In particular, impaired rotation resulted in random *situs inversus* or *solitus* (Okada et al., 1999, 2005), and experimentally reversed fluid flow caused *situs inversus* (Okada et al., 1999, 2005). Noteworthy from this work is that the *iv* gene itself did not code for left and right, but rather left and right are implicit in *situs solitus*, and the *iv* mutant merely disrupts that normal development, removing control, and leaving a random, 50:50, mixture of *situs solitus* and *situs inversus*. On that basis, *situs solitus* results from the asymmetric rotation of the cilia themselves, which presumably is due to them being built from *L*- rather than *D*-amino acids. In the unlikely future event that an organism could be created entirely from *D*-amino acids (and *L*-sugars), then presumably it would show *situs inversus*. Genes themselves therefore do not code asymmetry, but control the expression (or not) of pre-existing asymmetries from a lower level for which asymmetry was not primarily controlled genetically. Those worried about why amino acids and sugars in living organisms themselves show such large asymmetries are directed to the inevitably speculative final chapter of *Right Hand, Left Hand* (McManus, 2002).

Needless to say, it was hoped that handedness would be determined by the same ciliary mechanism as determined *situs*. However, it had been known for a while that humans with *situs inversus* had similar rates of left-handedness as did those with the heart on the left-hand side (Cockayne, 1938; Torgersen, 1950), and in 2004, that was confirmed to be the case in primary ciliary dyskinesia itself (McManus et al., 2004). Visceral asymmetry and brain asymmetry are therefore at least in part separately determined, although it seems more than probable that the underlying molecular machinery is conserved. There is though a recent suggestion that non-PCD *situs inversus* may show reversed or randomised handedness (Vingerhoets et al., 2018).

The 1990s also had their false lead, which inevitably consumed the energy of many researchers. In 1988, Diane Halpern and Stanley Coren published a short letter in *Nature* which asked, ‘Do right-handers live longer?’ (Halpern and Coren, 1988). Inevitably the answer was Yes – or as it was to be phrased repeatedly in newspapers, left-handers die 7 years earlier than right-handers. The data used came from an encyclopaedia of baseball players, and an eccentric usage of the Kolmogorov–Smirnov statistic apparently gave support to the claim. A continuing mystery is why *Nature* ever published this paper since the mean age at death of right-handers (64.64; standard deviation (SD) = 15.5, n = 1472) was clearly not different from that of left-handers (63.97; SD = 15.4, n = 236), as any competent first-year undergraduate could have shown using a t-test. Inevitably the paper provoked a world-wide response in newspapers. An even more provocative claim came in a letter to the *New England Journal of Medicine* (Halpern and Coren, 1991) which looked at the age at death of 987 Californians and found the left-handers died 9 years earlier (66 vs 75 for right-handers). Epidemiologists responded in droves, pointing out the difficulties of interpreting ‘death cohorts’, and more crucially they quoted data from prospective studies where age at death was clearly the same in right and left-handers. A lengthy critical review by Lauren Harris (1993b) also provoked extended response and counter-response (Halpern and Coren, 1993; Harris, 1993c), but despite this the idea became a cultural meme, what now would be called a ‘false fact’, and thanks once again to the Internet it appears to be completely resistant to evidence and argument. If further empirical evidence of its falsity is needed, the UK Biobank during 2006–2010 enrolled 500,000 people in a prospective study of those aged 40–69 (including this author). Within 5 years, about 8500 had died (Ganna and Ingelsson, 2015), and the relative mortality for left-handers, compared with right-handers, was almost exactly 1.0 (http://www.ubble.co.uk/association-explorer/). Left-handers undoubtedly do not die earlier, although the myth that they do appears to be immortal.

## The long 2000s

The long 2000s, with a little stretching, can be extended until the present day, and several major themes can be spotted, some beginning earlier but only becoming established in the 2000s.

Most earlier handedness research had been on humans, but comparative research across a wide range of species has been growing so that in 2007 it was a pleasure to welcome Giorgio Vallortigara as a co-editor of *Laterality.* Major contributions to the field have come from Lesley Rogers and Richard Andrew (2002) and have recently been reviewed by Rogers et al. (2013). There seem to be broad communalities in patterns of lateralisation across many species, although it is still open to debate whether human laterality is merely a development of that found in many other vertebrate species, or whether there is an additional saltatory event to reach human laterality, as, for instance, Tim Crow (2003) has forcefully argued. Handedness has been looked at in detail in two particular groups of animals, mice and primates.

In mice, the early work of Collins (1968, 1969, 1975) has been extended and elegantly developed over a long period by Fred Biddle and Brenda Eales. Their genetic dissection of paw preference, looking at mice of different strains reared in symmetric and asymmetric worlds, has shown how short-term and long-term memory result in a gradual learning and also an adaptability that is perhaps under the control of two genetic loci (Biddle and Eales, 2006; Ribeiro et al., 2011, 2014).

As the closest relatives to humans, the great apes are inevitably of interest in studying handedness. Early work on chimpanzees had suggested a 50:50 mix of right- and left-hand preference, but that became controversial. The meta-analysis of Hopkins (2006) suggested that there was a right-ward predominance in the great apes, although the results are far from secure, as much showing the problems of the field, as finding indisputable results. A funnel plot suggested there is publication bias so that, as Rich Palmer (2002) had put it, the evidence ‘seems inconsistent and contradictory [and] … remains equivocal’. If there is a population bias, it is closer to a 65:35 ratio than the 90:10 ratio found in humans. An added concern involves the influence of captivity, studies of wild-living chimpanzees showing equal rates of right- and left-handedness (Marchant and McGrew, 1996), although greater use of one hand, irrespective of whether it was right or left, did result in more efficient foraging (McGrew and Marchant, 1999), emphasising Adam Smith’s dictum that it always pays to specialise.

Despite the inevitable interest in the great apes, handedness in the primates more generally is also important, and studies suggest a key role for ecology, with arboreal and terrestrial species differing in hand usage (Meguerditchian et al., 2013), which is compatible with the postural origins theory of handedness, developed in the 1980s (MacNeilage, 2007; MacNeilage et al., 1987). The role of ecology was also emphasised in the first study of seven species of marsupials (Giljov et al., 2015) where there was overall a *left-hand* preference. While population lateralisation was present for the bipedal species, it was absent in quadrupedal species (such as Goodfellow’s tree kangaroo). The patterns of handedness do not relate to phylogeny (the three terrestrial kangaroo species all being left-handed), suggesting again the importance of ecological factors in handedness. Clearly, there is much of interest to be found in systematic studies of other species.

The use of meta-analysis in the study of handedness in great apes was part of a broader tendency for meta-analysis to resolve issues within laterality research in the past two decades, although it had been used earlier. A particularly good example is the association of handedness with male homosexuality and/or HIV/AIDS infection. Many studies in the 1980s and 1990s had asked whether there was any relationship with handedness, and we, like others, had concluded that there was not (Marchant-Haycox et al., 1991). However, a meta-analysis of 20 studies, including our own, with a grand total of 6182 homosexual men altered that situation, male homosexuals having a significantly higher likelihood of being left-handed (Lalumière et al., 2000). The earlier studies had mostly failed through being too small, a typical study having about 300 homosexual men, and hence being under-powered. That was later confirmed in the large BBC Internet study of sex and sexuality, where 4616 male homosexuals showed a significant excess of left-handedness (Blanchard and Lippa, 2007), with a similar effect found in the 2008 female homosexuals.

Sex differences in general have long been apparent in studies of handedness, males being more likely to be left-handed than females, as was shown in a meta-analysis of nearly 1,800,000 participants in 141 studies, where the odds ratio for a male being left-handed was 1.23 (95% confidence interval (CI) = 1.19–1.27) (Papadatou-Pastou et al., 2008). Despite the ubiquity of the sex difference, there are at present no proper explanations of why for every four left-handed females there should be five left-handed males. Attempts to create X-linked genetics models (Jones and Martin, 2010) have generally failed since they predict effects which are far larger than those actually found (McManus, 2010), although it is possible that autosomal modifier genes may be involved (McManus and Bryden, 1992).

Sex differences also resulted in a clear false lead which misled researchers, when it was suggested (once again in a paper in *Nature*) that there was ‘clear evidence for a sex difference in the functional organisation of the brain for language’, the cover picture that week showing functional magnetic resonance imaging (fMRI) scans with phonological processing entirely in the left hemisphere in males and organised bilaterally in females (Shaywitz et al., 1995). The study was though based on only 38 participants, and a later meta-analysis of 29 studies using 2151 participants found no sex differences (Sommer and Kahn, 2009). The meta-analysis also found no sex differences in planum temporale asymmetry, which is often thought to be related to language lateralisation, or in the right-ear advantage in dichotic listening. Although there appears to be no sex difference in lateralisation for language, that does leave a difficult theoretical problem as there is a robust sex difference in handedness, and most models assume that the same underlying genetic processes determine handedness and language lateralisation, and it is unclear why there should not also be a sex difference in language lateralisation (McManus, 2010). This particular false lead has therefore led in this case to an important question that is yet to be resolved.

Perhaps unsurprisingly the long 2000s is the decade both of fMRI scanning and molecular genetics. On the genetic front, high throughput processing of SNPs (single nucleotide polymorphisms) meant that large numbers of individuals could take part in GWASs (genome-wide association studies). Very few, however, looked at handedness and those that did were not published properly, being tagged on to existing studies, with only a single abstract published which described such work (Medland et al., 2009), but the net result had no significant associations. That result was made more solid by work carried out in conjunction with colleagues in Nottingham, where a GWAS not only showed no association with handedness but also, and importantly, that there was more than adequate power to detect any locus of a single-gene model (Armour et al., 2014). Although that might seem to be death knell for simple genetics, in fact it is not. If there are multiple genetic loci affecting different locations in a single biological ‘chain’ (and we estimated perhaps 40 or more), the dysfunction can be similar despite the multiple causes, as seems to be the case for primary ciliary dyskinesia (Fliegauf et al., 2007). A mundane example is that a car can show the same problem (failing to go), because of multiple different things wrong under the bonnet and in the transmission. Modelling multiple loci for handedness finds that the predicted patterns in families, twins and in relation to language dominance, are actually barely changed from a single-gene model (McManus et al., 2013).

Although neuroimaging had been used for studying brain activity since the late 1980s, most early studies used relatively small numbers of participants, they averaged results and most problematically, they tended to use only male right-handers. The result was that individual differences were impossible to study. That changed as fMRI became cheaper and more sensitive so that individuals could be scanned and compared with other individuals. A particularly important study is that of Badzakova-Trajkov and colleagues (2010), who studied 155 subjects, looking not only at the lateralisation of language processing but also at face processing and spatial attention. What became clear was that all possible combinations of language and face processing occurred in addition to the typical pattern of language on the left and face processing on the right, with atypical patterns more frequent in left-handers (a result first suggested by Bryden et al. (1983) on the basis of lesion studies). Genetic models have long postulated such differences in what I have called ‘cerebral polymorphisms’, the presumption being that they are generated by the genes determining handedness and lateralisation (McManus, 2009). Understanding variability in human cerebral organisation might, it is hoped, help in understanding a range of conditions associated with atypical laterality, such as dyslexia, stuttering, autism and schizophrenia, as well as perhaps illuminating special talents which have long been claimed to be related to left-handedness.

A separate advance in neuroimaging has been the development of functional transcranial Doppler (fTCD) for assessing cerebral dominance by comparing blood flow in the right and left middle cerebral arteries (Knecht et al., 1998, 2000a, 2000b). Relatively cheap, non-invasive, easily portable and practical in young children (Whitehouse et al., 2009), it is an ideal technique for large-scale studies, particularly as there seems to be a clear separation of right-hemisphere language from left-hemisphere language.

Surprisingly, the nature of handedness itself has been little looked at using fMRI, the self-evident difference between the two hands being studied surprisingly rarely, despite a general recognition that left-handers are less lateralised than are right-handers (McManus et al., 2016). An important study using fMRI is that of Nathalie Tzourio-Mazoyer and colleagues (2015) who studied 142 right-handers and 142 left-handers carrying out regular 2 Hz finger-tapping while in a scanner. The results are complex but suggest that in right-handers the dominant hemisphere inhibits the non-dominant hemisphere, but that occurs less in left-handers, and that there is variation of transcallosal inhibition in both right and left-handers. How and when that process occurs during development will inevitably be of interest.

Another aspect of how and why right- and left-handers differ in motor skill was raised for us by a forensic case where a murder seemed to have been committed using the left-hand but the defendant claimed to be right-handed (McManus et al., 2018). As well as needing to assess ‘true’ handedness, the study also forced questions of how well individuals can fake being of opposite handedness, and how and why individuals varied in at that ability. That raised interest in social cognitions of handedness – it seems that people vary in the ability to perceive the handedness of others, which in turn influences faking ability. Such issues inevitably lead onto the very neglected area of the phenomenology of handedness, on the lived experience of being right- or left-handedness, and on questions of identity (Westmoreland, 2017), all of which deserve further study.

## The next half-century

If five decades ago I could have seen what the next half-century would find about handedness, as well the false leads, I surely would have done my own research differently. To predict the future is hard, very hard, and as the physicist Niels Bohr is said to have remarked, ‘prediction is very difficult, especially about the future’ (Ellis, 1970: 431). If I have any clear sense of where things are going in lateralization, it is that large databases will help to unravel many of the issues, particularly where behavioural, social, genetic and neuroimaging data can be brought together, the UK Biobank being the paradigm for that, even if the phenotyping often far less sophisticated than the genotyping.

The promise of Biobank began to be realised in the autumn of 2018 when a host of genetic and neurological data was released by UK Biobank. Two studies found genetic loci that might be related to handedness (de Kovel and Francks, 2018; Wiberg et al., 2018), albeit with relatively small effects, with influences on microtubule proteins expressed in brain being a common theme, a finding that may well help to unpick some of the underlying biology. A separate part of UK Biobank is also brain scanning participants, and recently fMRI data have been released for 9000 participants (and in a few years that number should rise to 100,000). One of the genes linked to handedness seems also to relate to differences in white matter tracts linking together language areas (Wiberg et al., 2018), suggesting a link with the neuropsychology of language. These two studies have provided some hard and interesting leads which the next decade or two will surely explore further and which may well answer some of the difficult and interesting questions concerning the nature of handedness and cerebral asymmetries. Finally, UK Biobank has also announced that it is exome sequencing 50,000 participants, which may well allow rare mutations to be detected, which is of particular interest for handedness. The future therefore should be very interesting for handedness and lateralisation research.
